# Emergency Medicine Milestones Final Ratings Are Often Subpar

**DOI:** 10.5811/westjem.18703

**Published:** 2024-08-16

**Authors:** Diane L. Gorgas, Kevin B. Joldersma, Felix K. Ankel, Wallace A. Carter, Melissa A. Barton, Earl J. Reisdorff

**Affiliations:** * Ohio State University Wexner Medical Center, Department of Emergency Medicine, Columbus, Ohio; † American Board of Emergency Medicine, East Lansing, Michigan; ‡ Regions Hospital, Department of Emergency Medicine, St. Paul, Minnesota; § Weill Cornell Medicine, Department of Emergency Medicine, New York, New York

## Abstract

**Background:**

The emergency medicine (EM) milestones are objective behaviors that are categorized into thematic domains called “subcompetencies” (eg, emergency stabilization). The scale for rating milestones is predicated on the assumption that a rating (level) of 1.0 corresponds to an incoming EM-1 resident and a rating of 4.0 is the “target rating” (albeit not an expectation) for a graduating resident. Our aim in this study was to determine the frequency with which graduating residents received the target milestone ratings.

**Methods:**

This retrospective, cross-sectional study was a secondary analysis of a dataset used in a prior study but was not reported previously. We analyzed milestone subcompetency ratings from April 25–June 24, 2022 for categorical EM residents in their final year of training. Ratings were dichotomized as meeting the expected level at the time of program completion (ratings of ≥3.5) and not meeting the expected level at the time of program completion (ratings of ≤3.0). We calculated the number of residents who did not achieve target ratings for each of the subcompetencies.

**Results:**

In Spring 2022, of the 2,637 residents in the spring of their last year of training, 1,613 (61.2%) achieved a rating of ≥3.5 on every subcompetency and 1,024 (38.8%) failed to achieve that rating on at least one subcompetency. There were 250 residents (9.5%) who failed to achieve half of their expected subcompetency ratings and 105 (4.0%) who failed to achieve the expected rating (ie, rating was ≤3.0) on every subcompetency.

**Conclusion:**

When using an EM milestone rating threshold of 3.5, only 61.2% of physicians achieved the target ratings for program graduation; 4.0% of physicians failed to achieve target ratings for any milestone subcompetency; and 9.5% of physicians failed to achieve the target ratings for graduating residents in half of the subcompetencies.

## INTRODUCTION

With the advent of the Next Accreditation System (NAS), the Accreditation Council for Graduate Medical Education (ACGME) introduced a new assessment process called the “milestones.”^1^ The milestones are objective behaviors that reflect elements of the major competencies (eg, patient care, systems-based practice) in thematic domains called “subcompetencies” (eg, emergency stabilization, patient- and family-centered communication). The milestone scale uses nine ratings from 1.0, 1.5, 2.0, 2.5, etc, to 5.0. The scale is predicated on the assumption that a rating (level) of 1.0 corresponds to an incoming emergency medicine (EM)-1 resident and a rating of 4.0 is the graduation “target,” albeit not a graduation expectation or requirement. According to the ACGME: “Level 4 is designed as a graduation *goal* but *does not* represent a graduation *requirement*.”^2^ The EM milestones have been used exclusively as a formative assessment by the ACGME. Likewise, a physician’s EM milestone ratings are not considered when determining the eligibility of a physician to take the American Board of Emergency Medicine (ABEM) written qualifying examination.

The EM milestones were introduced in 2012, and the first ratings were reported in 2013.^3^ The EM milestones were revised in 2021, resulting in 22 subcompetencies. Since 2012, substantial validity evidence for the EM milestones has been accumulated.^4^ ^–^ ^10^ A resident’s milestone ratings are usually assigned by clinical competency committees (CCCs). Some subcompetency ratings are below target levels. Often, the subcompetency ratings assigned by the CCCs are lower than the ratings that residents give themselves.^11^ The milestones were initially designed to have a rating of 4.0 as the target for a resident completing an EM residency.^9^ Aggregate EM milestones are reported annually by the ACGME.^12^ These data and other reports suggest that a substantial number of graduating residents are not achieving a level 4 rating in many milestone subcompetencies.

We undertook this study to determine the frequency with which graduating residents received the target milestone rating.

## METHODS

### Study Design

This retrospective cross-sectional study was a secondary analysis of an already de-identified dataset used in a prior study.^13^ Our current study was deemed exempt from human subject research by the Western-Copernicus Group Institutional Review Board. The dataset available to the investigators did not include physician or program characteristics that would allow a more detailed analysis.

### Study Setting and Population

We analyzed milestone subcompetency ratings from Spring 2022 for categorical EM residents in their final year of training. These milestone ratings were submitted between April 25–June 24. This ratings report used EM Milestones 2.0, which included 22 subcompetencies. The dataset had been provided earlier to ABEM by the ACGME as part of the routine EM milestones secure data-sharing process.

### Measurements or Key Outcome Measures

The primary measure was the number of subcompetencies for which physicians failed to achieve a target rating of 3.5 at the time that the Spring milestone ratings were submitted to the ACGME. Because the ratings were submitted between April and June prior to residency completion, and the CCC could have determined the ratings even earlier than that, an expected rating for purposes of the study was modified to be 3.5 rather than 4.0. Doing so assumed that the resident would achieve a rating of 4.0 over the remaining weeks to months of residency training. We determined the number of physicians who did not achieve the target rating for the subcompetencies (from 0 subcompetencies to all 22 subcompetencies).

### Data Analysis

Ratings were dichotomized as meeting the target level at the time of program completion (≥3.5) and not meeting the target level at the time of program completion (≤3.0). We calculated the number of competencies for which a target rating was not achieved.

## RESULTS

In Spring 2022, there were milestone ratings for 2,637 residents in the Spring of their last year of training in 279 EM residencies. There were 1,613 residents (61.2%) who achieved a rating of ≥3.5 on every subcompetency and 1,024 residents (38.8%) who failed to achieve a rating of ≥ 3.5 on at least one subcompetency. There were 250 physicians (9.5%) who failed to meet half of their target subcompetency ratings. There were 105 residents (4.0%) who failed to meet the target rating (ie, rating was ≤3.0) on every subcompetency (Table).

**Table.** The frequency of emergency medicine residents receiving target milestones ratings lower that 3.5 in Spring 2022 (n = 2,637).

Number of ratings lower than 3.5	Number of terminal-year residents	Percent of total
0	1613	61.2
1	235	8.9
2	155	5.9
3	97	3.7
4	77	2.9
5	68	2.6
6	39	1.5
7	35	1.3
8	21	0.8
9	22	0.8
10	15	0.6
11	10	0.4
12	15	0.6
13	16	0.6
14	19	0.7
15	12	0.5
16	9	0.3
17	15	0.6
18	11	0.4
19	19	0.7
20	14	0.5
21	15	0.6
22	105	4.0

## LIMITATIONS

First, the actual level of subcompetency achievement at graduation was imprecisely known. We chose a rating of ≥3.5 to represent the performance target, given that the milestone ratings were provided prior to the completion of the program. Using a rating of 4.0 to be assigned two months prior to graduation would likely underestimate subcompetency achievement and a score of 3.5 at two months prior to program completion would likely overestimate subcompetency achievement. Anticipating that all residents with a rating of 3.5 would achieve a rating of 4.0 within weeks was a benevolent assumption. Second, demographic data on residents (eg, gender) and program characteristics (eg, duration of training) were unavailable to the investigators. Although this lack of additional information limited our ability to determine factors associated with the ratings, we believe that the findings are sufficiently significant on their merit and warrant additional investigation.

Third, we did not correlate poor subcompetency ratings with program extension or remediation, thus limiting the opportunity to gather any evidence of predictive or consequential validity. It is possible that nearly every physician who did not achieve a rating of ≥3.5 on nearly half of the milestone subcompetencies underwent remediation. Fourth, the ratings are assigned by CCCs. The structures of, and information used by CCCs, vary by EM residency.^14^ ^,^ ^15^ We did not attempt to determine the reliability or accuracy of the individual ratings. Moreover, we did not examine the potential impact of bias in the ratings. Prior studies suggested that women were assigned lower performance ratings.^16^ ^,^ ^17^ Sixth, the ratings used for this study were from the first year of the EM Milestones 2.0. Although there was a degree of acclimation in developing facility with the EM Milestones 1.0, it is likely that the same degree of unfamiliarity would be less with the most recent version. The degree to which the continued use of EM Milestones 2.0 will change rating trends is unknown.

## DISCUSSION

This study is the first in EM to demonstrate the degree to which physicians completing EM residencies are not achieving target subcompetency ratings. These data showed that of the 2,637 residents in their last year of training, nearly one in ten failed to meet target ratings for half of the EM subcompetencies. A similar finding was reported for physicians completing pediatric EM fellowships.^18^ However, that report used a target rating of 4.0, not 3.5 as in our study. Consequently, 67% of pediatric EM fellows did not attain a rating of at least 4.0 for at least one subcompetency.

A physician should be able to graduate from residency without scoring 4.0 on all 22 subcompetencies. In fact, all 4.0 ratings (a straight-line score) would be highly improbable.^19^ Consider the hypothetical situation that would result from the milestones being used in a summative manner to determine ABEM board eligibility. If residents were required to have no more than six subpar (ie, <3.5) milestone ratings (more than one-fourth of the subcompetencies), then 353 residents (13.4%) in their final year of training would not be eligible to take the ABEM written qualifying examination. Given the intent of the milestones as a formative instrument, ABEM maintains the position that the milestones should not be used as a summative determinant of board eligibility.

The rate of program extension by physicians beyond a scheduled graduation date has been reported to be approximately 8.5%.^13^ These extensions include physicians undergoing academic remediation, as well as program extensions due to a personal leave of absence. The prevalence of physicians not meeting half of the target subcompetency ratings was 9.5%. Based on these findings, there were physicians who failed to meet at least half of the EM milestone subcompetencies yet were deemed competent to practice autonomously as attested by the program director. This likelihood does not challenge the construct validity of the milestones, nor does it suggest that the target is too high. In a fact, a prior validity study by Korte et al used program director survey data to verify the appropriateness of the target ratings.^9^

In this study we did not analyze the impact of training length (EM1-3 vs EM1-4). However, a review of mean scores was undertaken in a prior investigation that used the same study period.^13^ The scores suggest that residents in EM1-3 programs tended to have higher scores through the postgraduate years (PGY) 1–3. For example, in the PGY-3 year, residents from EM1-3 programs had a mean rating of 3.51 (95% confidence interval [CI] 3.50–3.53) and residents from EM1-4 programs had a mean rating of 3.07 (95% CI 3.05–3.09), while EM4 residents had a mean rating of 3.67 (95% CI 3.65–3.69).

This analysis is an initial exploration into a more thorough investigation of the final milestones rating that an EM resident receives. The current study does not identify variable impact within demographic groups, nor does it provide any indices of predictive validity. Given the findings of this analysis, a more thorough analysis of the milestones should be undertaken to determine their psychometric qualities and subsequent utility in the field. Given the use of the milestones as a formative evaluation system, it should not be used to make summative decisions such as the determination of ABEM board eligibility. A more structured, valid, and reliable process for making the summative determination that a physician has demonstrated the necessary competencies to practice safely and independently is advisable. Moreover, such a detailed summative process could also be used to make a confident determination that a physician is eligible for board certification. This process would be easily accommodated in a model of competency-based medical education.

## CONCLUSIONS

Many physicians complete an EM residency without meeting a target rating for a graduating resident in up to half of the EM milestones. Some residents (4%) did not meet a target rating in any milestone. These findings support the continued use of the milestones as a formative instrument, rather than a tool to determine board eligibility.
